# Illuminating the *FGFR* fusion landscape in Chinese patients: unveiling novel molecular insights and clinical implications

**DOI:** 10.1093/oncolo/oyaf347

**Published:** 2025-10-14

**Authors:** Zhuo Liu, Dandan Zhao, Baoming Wang, Zhenyuan Qian, Zhengchuang Liu, Qiong Yang, Jinhua Tao, Yanxi Shao, Min Lv, Yanxiang Zhang, Jianhua Zhu, Jie Zhang, Wei Li, Xiaojuan Wang, Chunyang Wang, Tonghui Ma, Yuping Zhu

**Affiliations:** Zhejiang Cancer Hospital, Institute of Basic Medicine and Cancer (IBMC), Chinese Academy of Sciences, Hangzhou, Zhejiang 310022, China; College of Life Sciences, Jilin Agriculture University, Changchun 130118, China; Jichenjunchuang Clinical Laboratory, Hangzhou 310000, China; Jichenjunchuang Clinical Laboratory, Hangzhou 310000, China; General Surgery, Cancer Center, Department of Gastrointestinal and Pancreatic Surgery, Zhejiang Provincial People’s Hospital (Affiliated People’s Hospital, Hangzhou Medical College), Hangzhou 310014, China; Cancer Center, Key Laboratory of Tumor Molecular Diagnosis and Individualized Medicine of Zhejiang Province, Zhejiang Provincial People’s Hospital, Affiliated People’s Hospital, Hangzhou Medical College, Hangzhou 310014, China; Cancer Center, Clinical Research Center, Zhejiang Provincial People’s Hospital (Affiliated People’s Hospital, Hangzhou Medical College), Hangzhou 310014, China; General Surgery, Cancer Center, Department of Breast Surgery, Zhejiang Provincial People’s Hospital (Affiliated People’s Hospital, Hangzhou Medical College), Hangzhou 310014, China; Zhejiang Cancer Hospital, Institute of Basic Medicine and Cancer (IBMC), Chinese Academy of Sciences, Hangzhou, Zhejiang 310022, China; Zhejiang Cancer Hospital, Institute of Basic Medicine and Cancer (IBMC), Chinese Academy of Sciences, Hangzhou, Zhejiang 310022, China; Zhejiang Cancer Hospital, Institute of Basic Medicine and Cancer (IBMC), Chinese Academy of Sciences, Hangzhou, Zhejiang 310022, China; Jichenjunchuang Clinical Laboratory, Hangzhou 310000, China; Hangzhou Cosmos Wisdom Biotechnology Co. Ltd, Hangzhou 311215, China; Jichenjunchuang Clinical Laboratory, Hangzhou 310000, China; Jichenjunchuang Clinical Laboratory, Hangzhou 310000, China; Jichenjunchuang Clinical Laboratory, Hangzhou 310000, China; Jichenjunchuang Clinical Laboratory, Hangzhou 310000, China; Jichenjunchuang Clinical Laboratory, Hangzhou 310000, China; Genecn-Biotech Co. Ltd, Hangzhou 310000, China; Zybio Inc, Chongqing 400000, China; Zhejiang Cancer Hospital, Institute of Basic Medicine and Cancer (IBMC), Chinese Academy of Sciences, Hangzhou, Zhejiang 310022, China

**Keywords:** FGFR, rearrangements, molecular profiling, Chinese population, NGS

## Abstract

**Background:**

Despite the increasing approval and ongoing clinical trials of *FGFR*-targeted therapies, accurately detecting *FGFR* fusions remains a challenge due to limited research, low incidence rates, complex fusion partner distribution, and unique kinase domain distribution.

**Methods:**

We conducted a multicenter study to comprehensively profile *FGFR* fusions in the largest Chinese pan-cancer cohort to date, comprising 118 *FGFR* fusions from 114 individuals. Both DNA- and RNA-based sequencing approaches were utilized to reveal novel and fundamental features of *FGFR* fusion.

**Results:**

Our research reveals an incidence rate of 0.96% for *FGFR* rearrangements within this Chinese cohort, including a high incidence rate of *FGFR* fusions (40%) in parotid gland carcinoma. However, this is based on a small sample size of 5 tumors and should be interpreted cautiously pending validation in larger cohorts. We also uncovered distinct breakpoint distribution patterns across various *FGFR* rearrangements. For example, a primary breakpoint in intron17 of *FGFR2* was predominant (21/22), while *FGFR1/3* breakpoints displayed substantial diversity. For the first time, we identified “hot” breakpoints in *FGFR1* intron17, exon18, and *FGFR3*'s 3’ untranslated region. These findings underline the importance of incorporating these regions in targeted sequencing to ensure comprehensive detection of *FGFR1/3* fusions. Notably, we observed a predilection for intrachromosomal distribution in common *FGFR1/2/3* fusions. In contrast, most novel fusions (12/15) exhibited an interchromosomal distribution pattern, indicating variations in the fusion formation mechanism. Importantly, our study demonstrates the substantial incremental value of RNA-NGS or other orthogonal methods in confirming the functionality of *FGFR* rearrangements initially identified by DNA sequencing. In our cohort, 46% (6/13) of rare *FGFR1/2/3* fusions lacked detectable RNA transcripts; however, this does not definitively indicate non-functionality as factors such as low RNA quality, expression below detection limits, or nonsense-mediated decay may contribute. Therefore, RNA-based validation is critical for accurately identifying potentially targetable *FGFR* fusions and guiding therapy.

**Conclusion:**

Our findings offer critical novel insights into functional *FGFR* fusions and bear considerable clinical implications for identifying individuals whose tumors are most likely to respond favorably to *FGFR*-targeted therapies.

Implications for practice This study unveils the intricate landscape of *FGFR* fusions in Chinese patients with pan-solid tumor, offering novel molecular insights. Integration of DNA- and RNA-based sequencing uncovers a rare 0.96% incidence rate of *FGFR* rearrangements, notably prevalent in parotid gland cancer. Identification of distinct breakpoint patterns and “hot” breakpoints in *FGFR1/3* illuminates fusion formation mechanisms, crucial for comprehensive detection and offering valuable guidance for optimizing next-generation sequencing (NGS) panel designs to enhance sensitivity in identifying *FGFR* rearrangements for the first time. Emphasizing RNA-NGS validation enhances accuracy in identifying functional *FGFR* rearrangements, advancing precision medicine and patient management strategies.

## Introduction

The fibroblast growth factor receptor (*FGFR*) tyrosine kinase gene family has attracted considerable attention as a valuable target in cancer therapy.^1–3^ Specifically, U.S. Food and Drug Administration (FDA)-approved targeted therapies such as erdafitinib, pemigatinib, and infigratinib are employed for treating patients with *FGFR* genetic alterations in bladder cancer or cholangiocarcinoma.^4–6^ However, the accurate diagnosis of oncogenic *FGFR* rearrangements—a significant type of *FGFR* gene alteration—remains challenging due to their low frequency, complex distribution of fusion partners, and limited research.^7–13^

Previous studies, constrained by sample size and cancer type diversity, have reported inconsistent *FGFR* fusion distributions within the same tumor types across different cohorts.^7–10^ This inconsistency could be due to biases stemming from restricted sample sizes in unique cohorts. For example, *FGFR* fusions were absent in cervical cancer and melanoma in the Memorial Sloan Kettering Cancer Center (MSKCC) cohorts, in contrast to a Chinese cohort that reported *FGFR* fusions in over 10% of these cases. Conversely, the MSKCC cohorts showed a higher incidence of *FGFR* fusions in colorectal cancer, endometrial cancer, and glioma.^7^^,^^8^

Drawing on our experience in characterizing oncogenic gene fusions such as *ALK*, *ROS1*, *RET*, and *MET*,^9–17^ which have highlighted the complexity and challenges of fusion detection, we recognized that similar issues exist for *FGFR* fusions. Unlike widely researched fusions where the kinase region is typically located at the 3’ end, *FGFR* fusions may have their kinase region at either the 3’ or 5’ end. This peculiarity may result in *FGFR* fusions displaying characteristics markedly different from other fusions such as *ALK*, *ROS1*, and *RET*. However, the fundamental profiles of *FGFR* fusions, such as breakpoint distribution or fusion partner characteristics, remain poorly understood. Moreover, there is a lack of studies on the differences in *FGFR* fusion detection at the DNA and RNA levels. These research gaps could greatly significantly *FGFR* accuracy of detection and the efficacy of *FGFR* inhibitors.

In this study, we conducted a multicenter investigation of the molecular profiling of *FGFR* fusions in the largest Chinese pan-cancer cohort to date, integrating both DNA- and RNA-based sequencing. Our findings provide vital insights into functional *FGFR* fusions and hold significant clinical implications for identifying patients whose tumors are most likely to respond favorably to *FGFR*-targeted therapies.

## Materials and methods

### Patients and samples

Tumor samples from patients with pan-solid tumors, including glioma (GBM), head and neck carcinoma (HNC), lung carcinoma (LC), breast carcinoma (BRCA), gastric cancer (GC), colorectal carcinoma (CRC), bile duct carcinoma (BDC), hepatocellular carcinoma (HCC), kidney renal clear cell carcinoma (KIRC), prostate adenocarcinoma (PRAD), endometrial carcinoma (EC), cervical carcinoma (CC), melanoma (MC), soft tissue sarcoma (STS), and other cancer types, were collected between December 2018 and August 2022 for NGS-based *FGFR* fusion detection. Fresh tissue specimens were treated with RNAlater solution (Thermo Fisher Scientific) and cryopreserved at −80 °C or processed into formalin-fixed paraffin-embedded (FFPE) blocks, stored at 4 °C. FFPE blocks were prepared following previously described methods for nucleic acid recovery from FFPE tissue.^18^ The research was approved by the Ethics Committee of Zhejiang Provincial People’s Hospital (QT2022411) and adhered to the Helsinki Declaration.

### DNA sequencing

Genomic DNA was extracted from FFPE samples or frozen tissues, fragmented (150-200 base pairs), and analyzed using an 825-gene panel (Onco PanScan) targeting crucial tumor-related genes, as described in previous study.^17^ Quality control measures included removal of adapters and low-quality regions (Trimmomatic v0.36), aligning reads to the hg19 genome (Burrows-Wheeler Aligner v0.7.10), and assessing structural variations (GeneFuse v0.6.1, https://github.com/OpenGene/GeneFuse). Variants with a population frequency >0.1% were excluded based on Exome Aggregation Consortium guidelines, and the remaining variants were annotated using Oncotator and Variant Effect Predictor. *FGFR* fusion partners were classified based on their occurrence across 3 cohorts (our cohort, MSKCC 2021 and MSKCC 2017 cohorts), with those appearing in ≥ 3 samples designated as common, and those in 1-2 samples as uncommon.

### RNA sequencing

A 395-gene RNA panel (Fusioncapture) was utilized to detect *FGFR* fusions at the transcript level. Total RNA with RNA integrity DV200 ≥ 30% was reverse-transcribed into complementary DNA (cDNA), which was then sequenced on the Illumina NovaSeq 6000 platform. Reads were mapped to the human reference genome (hg19) using Hisat2-2.0.5,^19^ and gene fusions were identified with FusionMap.^20^

### Fluorescence in situ hybridization (FISH) and sanger sequencing

FISH and Sanger sequencing were used to validate novel fusions identified by the Onco PanScan and Fusioncapture panels. FISH was performed on FFPE sections using gene-specific probes, with 200 nuclei per probe analyzed. Sanger sequencing involved cDNA generated from 2.5 μg of total RNA, and fusion-specific primers were used for the polymerase chain reaction (PCR) amplification process. Reference sequences for fusion gene transcripts were obtained from the National Center for Biotechnology Information Reference Sequence Database. PCR products were purified and sequenced, with fusion sequences aligned with those detected by the Fusioncapture panel.

### Statistical analysis

Statistical analyses were conducted using Fisher’s exact test, chi-square test (with or without correction) in GraphPad Prism software (v8.0.1), with significance was set at *P* < .05.

## Results

### Prevalence of *FGFR* fusions in solid tumors

**Figure 1.**
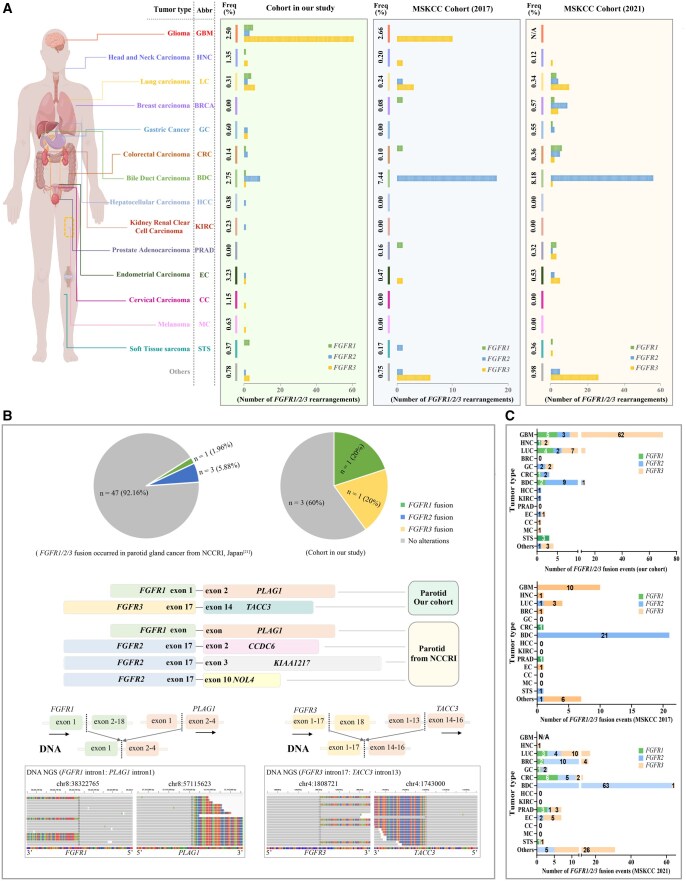
Comparison of prevalence and distribution of *FGFR* fusions in Chinese and MSKCC pan-solid tumor cohorts. (A) Bar charts of *FGFR* fusions frequencies across various tumor types in different cohorts. Schematic diagram of human tumor organs by Figdraw (https://www.figdraw.com/static/index.html) and bar charts of 118 *FGFR1/2/3* fusions from 114 pan-solid tumor patients in our cohort (left), 161 *FGFR1/2/3* fusions from 153 patients in the MSKCC 2017 cohort (middle), and 48 *FGFR1/2/3* fusions from 45 patients in the MSKCC 2021 cohort (right), respectively. (B) Pie Chart of *FGFR1/2/3* fusions harbored parotid gland cancer in the National Cancer Center Research Institute cohort (upper left) and in our cohort (upper right), respectively. Integrative Genomics Viewer (IGV) screenshots showing that *FGFR1-PLAG1* (bottom left) and *FGFR3-TACC3* (bottom right) in our parotid gland cancer patients, respectively. (C) Histogram of *FGFR1/2/3* fusions distribution in various cancer types among 3 cohorts. Our cohort (upper), MSKCC 2017 cohort (middle), and MSKCC 2021 cohort (bottom).

In our pan-solid tumor cohort of 11,898 patients, a total of 118 *FGFR* fusions were identified in 114 patients, including 16 *FGFR1* fusions (15 patients), 22 *FGFR2* fusions (22 patients), and 80 *FGFR3* fusions (78 patients), achieving an overall incidence rate of 0.96% (114/11,898) for cases with *FGFR* fusions-positive among all screened samples (Figure 1A and Table S1). The majority were found in GBM (60.53%, 69/114), LC (10.53%, 12/114), BDC (9.65%, 11/114) and GC (3.51%, 4/114). Notably, *FGFR* fusions prevalence in endometrial cancer was significantly higher than in the MSKCC cohorts (3.23% vs 0.53%; *P* = .0101), highlighting the potential clinical relevance and indicating a larger proportion of Chinese patients with EC whose tumors could potentially be candidates for *FGFR*-targeted therapies. Besides that, *FGFR* fusions not reported in the MSKCC cohorts were also identified in HCC, KIRC, CC, and MC, possibly due to population-specific differences. Formal statistical comparisons, however, were limited to tumor types with sufficient sample sizes (>10 cases) to avoid misleading conclusions. For tumor types with fewer than 10 cases, such as KIRC and CC, incidence rates are presented descriptively without statistical testing. No *FGFR* fusions were detected in breast or prostate cancer in our study, likely due to limited sample sizes (146 breast cancer and 123 prostate cancer). Interestingly, parotid gland cancer showed the highest *FGFR* fusion incidence 40% (2/5), far exceeding the 7.84% (4/51) reported in the National Cancer Center Research Institute cohort^21^ (Figure 1B). This finding implies a greater potential benefit from *FGFR* inhibitors for these individuals with such tumors. Furthermore, our cohort revealed that different types of *FGFR* fusions exhibit tumor-type specific preferences. For instance, *FGFR3* fusions dominated in glioma (91.43%) and lung cancer (53.85%), while *FGFR1* fusions were only observed in soft tissue sarcoma (Figure 1C and Table S2).

### Characterization of *FGFR* fusion partners in pan solid tumors

**Figure 2.**
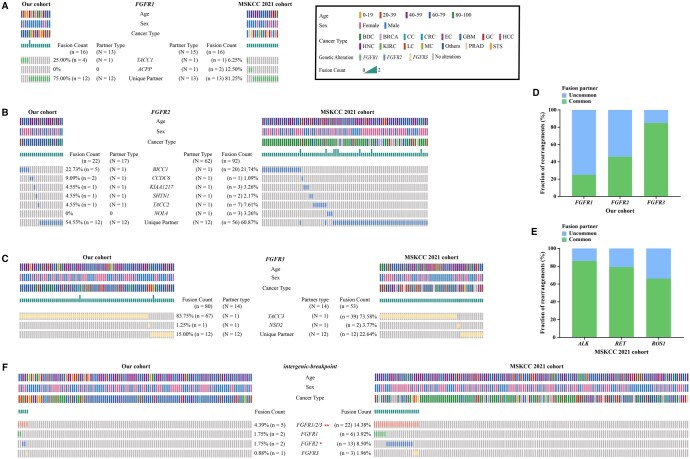
Molecular profiling of *FGFR1/2/3* fusions between Chinese and MSKCC 2021 cohorts. (A-C, F) Oncoprint heatmaps of *FGFR1* fusions (A), *FGFR2* fusions (B), *FGFR3* fusions (C), and intergenic-breakpoint *FGFR1/2/3* (F) in our cohort (left) and MSKCC 2021 cohort (right), respectively. Each column represents a patient. * and ** represents *P* < .01 and *P* < .01, respectively. (D, E) Histograms showed ratio statistics of “common” and “­uncommon” *FGFR1*, *FGFR2*, and *FGFR3* fusion partners in our cohort (D) and *ALK*, *RET* and *ROS1* fusion partners in MSKCC 2021 cohort (E).

A total of 44 unique partner genes were identified in the 118 *FGFR* fusions, indicating substantial heterogeneity. *FGFR1* (13/16, 81.25%) and *FGFR2* (17/22, 77.27%) showed diverse fusion types, with no dominant partner (Figure 2A-B), while *FGFR3* predominantly fused with *TACC3* (83.75%, 67/80) (Figure 2C), consistent with other classic receptor tyrosine kinase (RTK) rearrangements such as *ALK* (85.71%), *RET* (78.89%), and *ROS1* (65.70%) (Figure 2C-E). The similar pattern of *FGFR1/2/3* fusions was also observed in the other 2 MSKCC datasets (Figure S1A-E). Notably, *FGFR* intergenic-breakpoint fusions—novel potentially druggable alterations—were less common in our cohort (4.39%, 5/114) than in the MSKCC cohort (14.38%, 22/153), and included a newly identified *FGFR3* intergenic-breakpoint fusion in melanoma (Figure 2F and Table S3). Additionally, we identified 15 novel *FGFR* fusions with potential clinical significance racial differences across 6 cancer types, accounting for up to 13.16% (15/114) of cases (Table S3).

### Chromosomal distribution patterns of *FGFR* fusions

**Figure 3.**
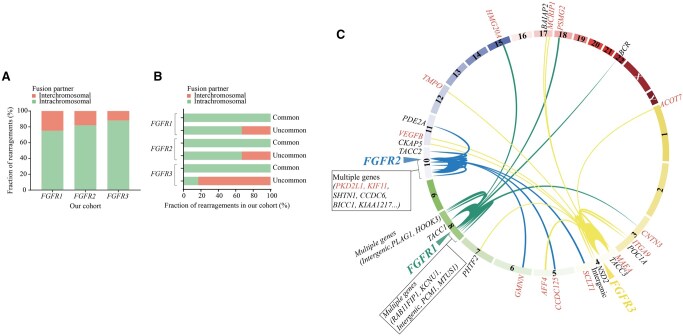
Chromosomal distribution patterns of *FGFR1/2/3* rearrangements. (A) Histogram showed proportions of *FGFR1/2/3* chromosomal rearrangements in our cohort. (B) Bar chart displayed the proportion statistics of “common” and “uncommon” *FGFR1/2/3* fusions chromosomal distribution. (C) Circos plots graphically depicting *FGFR1*, *FGFR2*, *FGFR3*, and their fusion partners in our cohort. Red font represents novel partners.

Chromosomal distribution is one of the important features of RTK rearrangements. Distinct chromosomal distribution patterns were observed among different RTK rearrangements: *ALK/RET* rearrangements mainly occurred intrachromosomally, while *ROS1* rearrangements predominantly clustered interchromosomally. Dissimilarities were also observed within the same kinase family, such as *NTRK1/2* (intrachromosomal) vs *NTRK3* (interchromosomal) (Figure S2A-D). In contrast to the above, we found for the first time that *FGFR1/2/3* fusions predominantly exhibited intrachromosomal distribution (75%, 81.82%, and 87.50%, respectively) (Figure 3A, Table S4), consistent with the MSKCC cohort (Figure S2E). However, interchromosomal fusions were mainly linked to novel partners (75% for *FGFR1/2* and 60% for *FGFR3*) (Table S4). Additionally, all common *FGFR1/2/3* fusion partners and most uncommon *FGFR1/2* partners (*FGFR1*: 8/12, 66.67%; *FGFR2*: 8/12, 66.67%) were intrachromosomal, whereas uncommon *FGFR3* fusion partners showed more interchromosomal distribution (10/12, 83.33%) (Figure 3B-C).

### Characterization of *FGFR1/2/3* fusion breakpoints

**Figure 4.**
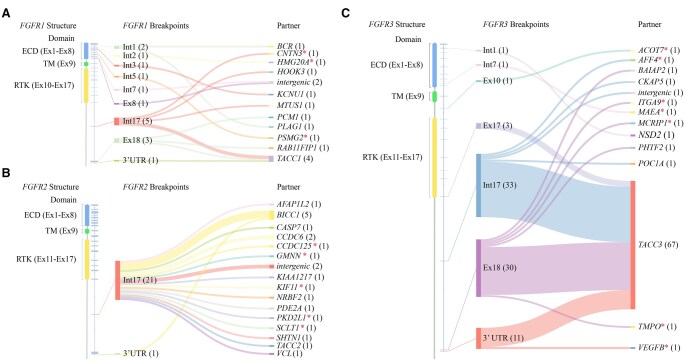
Distribution patterns of *FGFR1/2/3* fusion breakpoints by DNA-NGS. (A-C) Sankey diagrams showing the detecting results flow of 16 *FGFR1* (A), 22 *FGFR2* (B), and 80 *FGFR3* (C) rearrangements by SankeyMATIC (http://sankeymatic.com/build/). From left to right, the first columns representing the detailed structure domain of *FGFR1/2/3* genes, the middle columns showing breakpoints of *FGFR1/2/3* rearrangements, the right column displaying *FGFR1/2/3* fusion partners arranged in alphabetical order. Red asterisk * represents novel fusion partner. ECD, extracellular domain; TM, transmembrane domain; RTK, receptor tyrosine kinase.

Distinct breakpoint distributions were observed among *FGFR* rearrangements. *FGFR2* breakpoints predominantly clustered in intron17 (21/22, 95.45%), whereas *FGFR1* and *FGFR3* breakpoints were more dispersed across multiple introns (Figure 4A-C). Specifically, intron17 (5/16, 31.25%) and exon18 (3/16, 18.75%) were the 2 principal breakpoints for recurrent *FGFR1* rearrangements, while nearly all other dispersed breakpoints involved rare fusion partners (Figure 4A). For *FGFR3*, breakpoints primarily occurred in intron17 (33/80, 41.25%), exon18 (30/80, 37.50%), 3’ untranslated regions (UTR) (11/80, 13.75%) and exon17 (3/80, 3.75%) (Figure 4C). Notably, the predominant *FGFR1* breakpoints (intron17 and exon18), which together constituted half (8/16) of observed events, and specific *FGFR3* breakpoints (3’ UTR and exon17, 14/80 cases), were uniquely identified in our cohort compared to the MSKCC cohort (Figure 4A and Figure S3A; ­Figure 4C and Figure S3C). The discrepancy might be due to ethnic differences or the sensitivity and coverage of different sequencing panels (see Table S5 for detailed intronic bait coverage, including *FGFR1* intron 17). Additionally, significant intragenic regions preference between different *FGFR* rearrangements also existed. *FGFR1*/2 breakpoints mostly occurred in introns (68.75% 11/16 and 95.45% 21/22, respectively), while *FGFR3* breakpoints were more common in exonic regions (42.50%, 34/80) within the coding sequence (CDS) and the 3’ UTR (13.75%, 11/80) (Figure 4A-C), and similar trends were observed in the MSKCC cohort (­Figure S3A-C).

### RNA-based NGS validation and characterization of rare *FGFR1/2/3* fusions

Among the 118 *FGFR* fusions detected from 114 cases in our cohort, up to 17.54% (20/114) were rare, including 4.39% (5/114) intergenic-breakpoint fusions and 13.16% (15/114) novel fusions (Table 1 and Table S6). In total, of the 13 rare *FGFR* fusions that underwent RNA-NGS validation, 6 cases (46%) failed to generate a corresponding fusion transcript at the RNA level. This finding demonstrates that nearly half of the rare or novel *FGFR* fusions identified by DNA-NGS may be non-functional, highlighting the substantial incremental value of RNA-based validation. Functional validation of these rare fusions by RNA-NGS showed that 4 of 5 intergenic-breakpoint fusions were validated, with 2 intergenic*-FGFR1* fusions in lung cancer accounting for 50% of *FGFR1*-positive lung cases. However, these intergenic*-FGFR1* fusions were non-transcribable (Table 1 and Figures S4A-B and S5A-B). In contrast, the *FGFR2-*intergenic fusion in lung cancer produced a novel chimeric transcript with a complete kinase domain (*FGFR2-PLEKHA4,* E17: E10) (Figure S5C-D). The *FGFR2-PLEKHA4* fusion is potentially targetable, as it produces an in-frame transcript with an intact kinase domain confirmed by in silico domain mapping (Figure S6), though further functional validation is needed to confirm ligand-independent activation. The newly discovered *FGFR3-*intergenic fusion in melanoma also generated a functional transcript (*FGFR3-TNIP2*, E17: E2) (Figures S4C-5D). The functional bias observed among different *FGFR-*intergenic fusions underscores the need for further verification in clinical practice to guide targeted therapy more accurately.

**Table 1.** *FGFR1/2/3* rare fusions identified by DNA/RNA-based NGS.

Patient (ID)	Rare fusion type	Cancer type	DNA-NGS data				RNA-NGS data	
			Fusions	Breakpoints	In-frame/Out-frame	Intact fusion domain	Fusions	Breakpoints
Case 9	Intergenic-breakpoint fusions	LC	*intergenic-FGFR1*	*:Int7	N/A	Yes	Negative	Negative
Case 10		LC	*intergenic-FGFR1*	*:Int7	N/A	Yes	Negative	Negative
Case 19		GC	*FGFR2-intergenic*	Int17:*	N/A	Yes	N/A	N/A
Case 20		LC	*FGFR2-intergenic*	Int17:*	N/A	Yes	*FGFR2-PLEKHA4* ^a^	E17: E10
Case 40		MC	*FGFR3-intergenic*	Int17:*	N/A	Yes	*FGFR3-TNIP2*	E17: E2
Case 3	Novel fusions	LC	*FGFR1-HMG20A*	Int2: Int3	out-frame	No	Negative	Negative
Case 7		STS	*FGFR1-PSMG2*	Int5: Int2	out-frame	No	Negative	Negative
Case 37		GBM	*FGFR3-ACOT7*	E10: Int7	exon-intron	No	Negative	Negative
Case 46		LC	*FGFR3-VEGFB*	3'UTR: E5	3'UTR-exon	Yes	Negative	Negative
Case 25		BDC	*FGFR2-SCLT1*	Int17: Int2	in-frame	Yes	*FGFR2-SCLT1*	E17: E3
Case 21		GBM	*FGFR2-KIF11*	Int17: Int8	in-frame	Yes	*FGFR2-KIF11*	E17: E9
Case 38		GBM	*FGFR3-AFF4*	Int17: Int11	in-frame	Yes	*FGFR3-AFF4*	E17: E12
Case 41		STS	*FGFR3-ITGA9*	E18: Int27	exon-intron	Yes	*FGFR3-ITGA9*	E17: E28
Case 45		GBM	*FGFR3-TMPO*	E18: Int1	exon-intron	Yes	*FGFR3-TMPO*	E17: E2
Case 2		BDC	*CNTN3-FGFR1*	Int3: Int17	in-frame	No	N/A	N/A
Case 17		BDC	*FGFR2-CCDC125*	Int17: Int5	in-frame	Yes	N/A	N/A
Case 18		KIRC	*FGFR2-GMNN*	Int17: Int2	in-frame	Yes	N/A	N/A
Case 24		PAAD	*FGFR2-PKD2L1*	Int17: Int12	in-frame	Yes	N/A	N/A
Case 47		LC	*MAEA-FGFR3*	Int5: Int7	out-frame	Yes	N/A	N/A
Case 42		GBM	*FGFR3-MCRIP1*	E18: Int1	exon-intron	Yes	N/A	N/A

a Novel fusion was identified by RNA-NGS; in-frame transcript includes the full TKD coding region based on in silico domain mapping and is considered *potentially targetable*.

* Breakpoints of *FGFR* fusion partners were located in intergenic region.

Abbreviations: BDC, bile duct carcinoma; E, exon; GBM, glioma; GC, gastric cancer; Int, intron; N/A, not available; KIRC, kidney renal clear cell carcinoma; LC, lung carcinoma; MC, melanoma; PAAD, pancreatic adenocarcinoma; STS, soft tissue sarcoma.

Additionally, 9 novel *FGFR* rearrangements (2 *FGFR1*, 2 *FGFR2*, 5 *FGFR3*) with sufficient specimens were verified by RNA-NGS. Among these, 4 novel fusions were not expressed, while 5 generated chimeric coding transcripts (Table 1). Notably, 3 of the non-expressed fusions lacked intact *FGFR* kinase domains (Figure S5E-F), and one had a breakpoint after the *FGFR3* stop codon (Table 1 and Figure S4E-F). In contrast, among the 5 novel fusions with potential therapeutic targets for *FGFR* inhibitors, 3 in-frame *FGFR* fusions exhibited completely consistent forms at both DNA and RNA levels (Figure S5G-H and Figure S4G-H). However, discrepancies were observed in 2 exnoic *FGFR* novel samples: an *FGFR3* exon18-*ITGA9* intron27 fusion was detected at DNA level but underwent alternative splicing during transcription to form an in-frame *FGFR3* exon17-*ITGA9* exon28 fusion (Figure S5I-J), and a similar result was seen for *FGFR3-TMPO* fusion (E18: E2 to E17: E2) (Figure S4I-J). A subset of novel and rare fusions identified by NGS were further validated using orthogonal methods including FISH and Sanger sequencing, with representative results—such as those for the *FGFR2-SCLT1* fusion—shown in Figure S4H. These validation results were concordant with the NGS findings for the analyzed cases. Notably, in this study, no *FGFR1/2/3* fusions were identified exclusively by RNA-NGS that were not already detected by DNA-NGS, as our RNA analysis was limited to DNA-positive samples.

## Discussion

*FGFR1/2/3* have been recognized as oncogenic drivers contributing to tumorigenesis.^3^ However, comprehensive profiling of *FGFR* fusions remains limited. In this study, we analyzed the largest Chinese solid tumor cohort with *FGFR* fusions to date, identifying a total of 118 *FGFR* fusions and refining previously conflicting incidence rates of *FGFR* fusions in certain cancer types across different populations. Remarkably, Chinese patients with EC and parotid gland cancer displayed high ­clinical potential for targeted therapy. Additionally, the distribution of *FGFR* fusion breakpoints offers informative guidance for NGS panel design, with the predominant breakpoints of intron17 and exon18 in *FGFR1* fusions and the 3’UTR in *FGFR3* fusions revealed for the first time. Furthermore, functional transcripts were identified in rare *FGFR* fusions. However, inconsistencies were observed between DNA and RNA NGS in novel *FGFR* fusions with or without intact tyrosine kinase domain (TKD). In addition, functional preference was also observed among different *FGFR*-intergenic fusions. These findings provide valuable information for optimizing NGS to effectively diagnose low-frequency, diverse oncogenic *FGFR* fusions.

Compared to other RTK rearrangements such as *ALK* and *ROS1*,^7^^,^^22^  *FGFR1/2/3* rearrangements are notably less frequent in solid tumors, leading to a scarcity of clinical studies comprehensively identifying *FGFR* fusions. Previous research^23^^,^^24^ have preliminarily explored the profiling of *FGFR* fusions in different populations, and revealed a significantly higher incidence of *FGFR* fusions in Chinese patients compared to Caucasians in certain cancers, such as cervical carcinoma (12.5% vs 0) and melanoma (10% vs 0), and so on. However, the smaller cohort size in previous Chinese studies may have contributed to deviations. Our study included 11,898 Chinese pan-solid tumor cases, which, to our knowledge, represents one of the largest cohorts for *FGFR* fusion profiling reported in the literature (see Table S1 for detailed comparisons with previous studies). This large number of cases enabled us to provide more reliable incidence rates for certain cancer types, refining previous estimates from smaller Chinese cohorts while confirming the occurrence of *FGFR* fusions in Chinese patients with cervical carcinoma and melanoma.^23^^,^^24^ Additionally, *FGFR* fusions were exclusively identified in Chinese HCC and KIRC cases. These findings emphasize the specific cancer preferences of *FGFR* fusions across different populations, highlighting the potential benefits of *FGFR* inhibitors in various cancers among different ethnic groups. Moreover, our study confirmed for the first time that GBM in China is a dominant disease for *FGFR* rearrangements, similar to the MSKCC cohort.^8^ Strikingly, our study found a high *FGFR* fusion incidence of 40% in parotid gland carcinoma, but this result is based on a limited sample size of 5 patients, restricting statistical power and generalizability. Therefore, while suggestive of potential clinical benefit from *FGFR* inhibitors, these findings require validation in larger cohorts to confirm prevalence and clinical utility. These results suggest potential clinical utility of *FGFR* inhibitors in specific tumor types and highlight the need for further research to identify patient populations who may derive benefit.

The low incidence of *FGFR* fusions in solid tumors poses a major challenge for thorough detection,^25^ while the limited understanding of *FGFR* fusions’ molecular characteristics further compounds this issue. Comprehensive profiling of *FGFR* fusions to identify breakpoints and partners is necessary to optimize detection performance. Our study revealed that *FGFR2* fusions predominantly featured breakpoints in intron17 (∼3600 bp) (95.45%, 21/22), while *FGFR1*/3 exhibited highly diverse breakpoint distributions. Previous studies^7^^,^^24^ showed dispersed breakpoint characteristics in *FGFR1* fusions (introns 3-4, intron 10 and introns 15-16), whereas our study identified intron17 (∼3677 bp) (5/16, 31.25%) as a dominant breakpoint for the first time. This difference may be attributed to the panel coverage of intron17, which was fully covered in our study but not in the MSKCC-fusion panel. Additionally, exonic-breakpoint fusions were identified in 31.25% of *FGFR1* and 56.25% of *FGFR3* positive cases, indicating the importance of adequate exons coverage in targeted NGS panels for *FGFR1/3* fusions detection. Moreover, in contrast to other oncogenic rearrangements (*ALK*, *ROS1*, etc.),^26^^,^^27^ where breakpoints mainly distribute in the coding sequence (CDS), *FGFR* fusions, particularly in *FGFR3*, were often found in the 3′ UTR (13.75%, 11/80). This highlights that targeting only the CDS region during sequencing is not sufficient for detecting *FGFR* fusions; the 3′ UTR should also be considered in NGS panel design. Intuitively, incorporating the consequences of breakpoints distribution variations into sequencing assays can significantly improve *FGFR* fusions detection. However, due to extensively diverse breakpoint distributions and complex intron structures, off-target sequencing may occur, if only breakpoints are considered during panel design. Including major fusion partners is another strategy for effective detection. However, unlike *FGFR3*, which had a dominant partner (*TACC3*, 78.75%, 63/80), *FGFR1*/2 partners were more dispersed, with unique partners reaching up to 81.25% (13/16) and 77.27% (17/22), respectively. This suggests that including only *TACC3* maybe sufficient for enhancing *FGFR3* fusion detection, whereas a range of high-frequency partners is needed for *FGFR1*/2 fusions detection.

Although DNA-based NGS provides a broad approach for detecting *FGFR* rearrangements, our findings highlight significant discrepancies between DNA and RNA results, particularly for rare and novel fusions. In our cohort, nearly half (46%) of DNA-detected rare FGFR1/2/3 fusions could not be validated by RNA-NGS. While some of these events may indeed be non-functional or false positives, other factors such as degraded RNA, low transcript abundance, or post-transcriptional mechanisms (e.g., nonsense-mediated decay) could also explain the absence of RNA-level detection. Notably, the inability to detect RNA transcripts was especially evident among *intergenic-FGFR1* fusions and novel events lacking an intact kinase domain. Furthermore, RNA-level analysis revealed alternative splicing events that were not predictable from DNA data alone. These results underscore the substantial incremental value of RNA-NGS validation, which is essential for distinguishing clinically relevant, functional *FGFR* fusions and ensuring accurate patient selection for *FGFR*-targeted therapies. Based on these findings, we recommend that rare or atypical *FGFR* fusions identified by DNA-based assays undergo orthogonal validation prior to clinical decision-making. Recent studies^7^^,^^28^^,^^29^ have suggested that some intergenic-breakpoint fusions may be transcribed, but their functionality in *FGFR* fusions remains unclear, raising questions about the therapeutic potential of *FGFR* fusions with intergenic partners. For the first time, we found that functional preferences vary among different *FGFR*-intergenic fusions. Specifically, all intergenic-*FGFR1* fusions lacked RNA expression and were not clinically beneficial targets, consistent with previous findings.^28^ However, *FGFR2/3-*intergenic fusions generated functional fusion transcripts, broadening the potential therapeutic scope. This functional divergence suggests that *FGFR* subtype may influence outcomes of intergenic-breakpoint *FGFR* fusions, necessitating further studies for confirmation. More interestingly, unlike other oncogenic rearrangements such as *ALK*^9^ and *RET*,^26^ where rare genomic rearrangements can transform into canonical fusions, rare *FGFR* fusions exhibit similar forms at both DNA and RNA levels. This similarity may be attributed to the fact that the majority of rare genomic *ALK* and *RET* fusion partners are in close chromosomal proximity to their respective genes,^30^ whereas *FGFR* fusion partners do not exhibit this pattern. Additionally, our previous research^9^^,^^17^ showed that 5′-RTK fusions (5′-*ALK*, 5′-*RET*, etc.) can generate canonical functional transcripts even with intact TKD. Conversely, *FGFR* fusions, dominated by 5’ fusions, are not expressed when the TKD is incomplete. Such differences may be attributed to the structural characteristics of different oncogenic drivers. These novel findings emphasize the importance of validation assays, such as RNA NGS, for cases with intergenic-breakpoint or incomplete kinase domain *FGFR* fusions to guarantee the production of active chimeric proteins. A key limitation of our study is that, due to limited specimen availability and the low incidence of *FGFR* fusions, we were only able to perform RNA-based validation on a subset of DNA-positive cases. As a result, we could not determine whether RNA-NGS could identify additional *FGFR* rearrangements missed by DNA-based assays in this cohort. However, as demonstrated in our recent large-scale sarcoma cohort study,^31^ RNA-based sequencing can indeed uncover additional actionable fusions that are missed by DNA-NGS panels. Future comprehensive dual-platform analyses in solid tumors are warranted to fully evaluate this potential.

## Conclusion

Taken together, we conducted comprehensive molecular profiling of *FGFR*-positive fusions using one of the largest reported Chinese pan-solid tumor cohorts to date. Additionally, our results provide valuable insights for optimizing *FGFR* detection by NGS. Moreover, novel fusions were identified and validated, expanding the scope of *FGFR* rearrangements and providing important insights for clinical research and potential applications.

This study has several limitations. Despite observing a significant *FGFR* fusion rate in parotid gland cancer, the statistical power was constrained by the low incidence of this cancer type and the limited number of cases in our cohort. Therefore, a larger cohort size is required to further support our conclusions. Additionally, due to specimen unavailability, some *FGFR-*intergenic rearrangements identified by DNA NGS could not be validated by RNA NGS. It remains unclear whether intergenic-breakpoint fusions involving different *FGFR* genes (*FGFR1*, *FGFR2*, or *FGFR3*) consistently yield functional transcripts in larger patient cohorts. Further research is needed to clarify whether specific *FGFR-intergenic* rearrangements can generate transcripts that inform targeted therapeutic strategies. Moreover, further wet-lab work is needed to confirm the clinical value of novel *FGFR* fusions.

## Supplementary Material

oyaf347_Supplementary_Data

## Date availability

The data underlying this article will be shared on reasonable request to the corresponding author.
