# Supplementary material for: Illuminating the FGFR fusion landscape in Chinese patients: unveiling novel molecular insights and clinical implications
Source: Oncologist. 2025 Oct 14;30(11):oyaf347. doi: 10.1093/oncolo/oyaf347 (PMC12640125; doi:10.1093/oncolo/oyaf347)
Supplement: oyaf347_Supplementary_Data [file oyaf347_supplementary_data.zip › Supplementary Figure and Table legends.docx]

**Supplementary Figure and Table legends:**

**Supplementary Fig. S1. Molecular profiling of *FGFR1/2/3* fusions among Chinese and MSKCC 2017 cohorts.** (A-C) Oncoprint heatmaps of *FGFR1* fusions (A), *FGFR2* fusions (B), *FGFR3* fusions (C) in our cohort (left) and MSKCC 2017 cohort (right), respectively. Each column represents a patient. (D, E) Histograms showed ratio statistics of “common” and “uncommon” *FGFR1*, *FGFR2*, and *FGFR3* fusion partners in MSKCC 2017 cohort (D) and MSKCC 2021 cohort (E). N/A, not available.

**Supplementary Fig. S2. Chromosomal distribution patterns of *ALK*, *RET*, *ROS1*, *NTRK1/2/3*, and *FGFR1/2/3* rearrangements in MSKCC cohort.**

(A, B) Histograms showed percentages of *ALK*, *RET*, *ROS1*, and *NTRK1/2/3* in our cohort. (C-E) Histograms showed percentages of *ALK*, *RET*, *ROS1*, *NTRK1/2/3*, and *FGFR1/2/3* chromosomal rearrangements in MSKCC cohort.

**Supplementary Fig. S3. Distribution patterns of breakpoints in *FGFR1/2/3* fusion from MSKCC cohort.** (A-C) Sankey diagrams showing the detecting results flow of 16 *FGFR1* (A), 92 *FGFR2* (B), and 53 *FGFR3* (C) rearrangements by SankeyMATIC (<http://sankeymatic.com/build/>). From left to right, the first columns representing the detailed structure domain of *FGFR1/2/3* genes, the middle columns showing breakpoints of *FGFR1/2/3* rearrangements, the right column displaying *FGFR1/2/3* fusion partners arranged in alphabetical order. ECD: extracellular domain; TM: Transmembrane Domain; RTK: Receptor Tyrosine Kinase.

**Supplementary Fig. S4. Functional validation of genomic novel *FGFR* rearrangements by RNA-NGS.** (A-J) DNA/RNA IGV screenshots and schematic diagrams of genomic intergenic-breakpoint *FGFR1/3* in LC (A, B) and in MC (C, D), genomic 3’UTR-exon novel *FGFR3-VEGFB* in LC (E, F), genomic in-frame novel *FGFR2-SCLT1* in BDC (G, H), and genomic exonic-breakpoint novel *FGFR3-TMPO* in GBM (I, J). Blue, green, red, and orange blocks represent the ‘C’, ‘A’, ‘T’ and ‘G’ bases, respectively. BDC: Bile Duct Carcinoma; GBM: Glioma; LC: Lung carcinoma, MC: Melanoma.

**Supplementary Fig. S5. Functional validation of genomic rare *FGFR* rearrangements by RNA-NGS.** (A-J) DNA/RNA IGV screenshots and schematic diagrams of genomic intergenic-breakpoint *FGFR1/2* in LC (A-D), novel *FGFR1-PSMG2* without complete kinase domain in STS (E, F), genomic in-frame novel *FGFR2-KIF11* in GBM (G, H) and genomic exonic-breakpoint novel *FGFR3-ITGA9* in STS (I, J). Blue, green, red, and orange blocks represent the ‘C’, ‘A’, ‘T’ and ‘G’ bases, respectively. GBM: Glioma; LC: Lung carcinoma; STS: Soft tissue sarcoma. TKD, tyrosine kinase domain.

**Supplementary Fig. S6. Domain architecture analysis of novel fusion *FGFR2-PLEKHA4* detected by RNA-NGS**

**Supplementary Table S1. Frequency of *FGFR1-3* rearrangements in pan-solid tumors among Chinese and MSKCC cohorts**

**Supplementary Table S2. Proportion of *FGFR1/2/3* rearrangements across different cancer types in Chinese and MSKCC cohorts**

**Supplementary Table S3. Uncommon partners of *FGFR1-3* rearrangements by DNA-NGS in this study**

**Supplementary Table S4. Chromosome distribution of *FGFR1/2/3* rearrangements by DNA-NGS in our cohort**

**Supplementary Table S5. Coordinates and Coverage Summary of Intronic Bait Probes Targeting *FGFR1*, *FGFR2*, *FGFR3* and *FGFR4* in the DNA Panel**

**Supplementary Table S6. Chromosomal location for *FGFR1/2/3* rare fusions identified by DNA/RNA-based NGS**
