# Supplementary material for: Illuminating the FGFR fusion landscape in Chinese patients: unveiling novel molecular insights and clinical implications
Source: Oncologist. 2025 Oct 14;30(11):oyaf347. doi: 10.1093/oncolo/oyaf347 (PMC12640125; doi:10.1093/oncolo/oyaf347)
Supplement: oyaf347_Supplementary_Data [file oyaf347_supplementary_data.zip › Supplementary table S1.docx]

**Supplementary Table S1. Frequency of *FGFR1-3* rearrangements in pan-solid tumors among Chinese and MSKCC cohorts**

| **Cancer type** | **Abbreviation** | **Chinese cohort** | **MSKCC cohort (2017)** | **MSKCC cohort (2021)** | **Chinese cohort** | **MSKCC cohort (2017)** | **MSKCC cohort (2021)** | **Chinese vs. MSKCC (2017)** | **Chinese vs. MSKCC (2021)** |
| --- | --- | --- | --- | --- | --- | --- | --- | --- | --- |
|  |  | ***FGFR1-3* positive rearrangement (No.)** | | | ***FGFR1-3* rearrangement frequency (%)** | | | **P-value** | |
| Glioma | GBM | 69 | 10 | 0 | 2.50% | 2.66% | N/A | 0.8530^b^ | N/A |
| Head and Neck Carcinoma | HNC | 3 | 1 | 1 | 1.35% | 0.20% | 0.12% | 0.1649^c^ | ***0.0394^c^** |
| Lung Carcinoma | LC | 12 | 4 | 17 | 0.31% | 0.24% | 0.34% | 0.652^b^ | 0.8462^b^ |
| Breast Carcinoma | BRCA | 0 | 1 | 15 | 0.00% | 0.08% | 0.57% | >0.9999^a^ | >0.9999^a^ |
| Gastric Cancer | GC | 4 | 0 | 3 | 0.60% | 0.00% | 0.55% | 0.5777^a^ | 0.8055^c^ |
| Colorectal Carcinoma | CRC | 2 | 1 | 13 | 0.14% | 0.10% | 0.36% | 0.7301^c^ | 0.1901^b^ |
| Bile Duct Carcinoma | BDC | 11 | 18 | 57 | 2.75% | 7.44% | 8.18% | 0.0056^b^ | 0.0003^b^ |
| Hepatocellular Carcinoma | HCC | 1 | 0 | 0 | 0.38% | 0.00% | 0.00% | >0.9999^a^ | >0.9999^a^ |
| Kidney Renal Clear Cell Carcinoma | KIRC | 1 | 0 | 0 | 0.23% | 0.00% | 0.00% | >0.9999^a^ | >0.9999^a^ |
| Prostate Adenocarcinoma | PRAD | 0 | 1 | 7 | 0.00% | 0.16% | 0.32% | >0.9999^a^ | >0.9999^a^ |
| Endometrial Carcinoma | EC | 2 | 1 | 7 | 3.23% | 0.47% | 0.53% | 0.2566^c^ | ***0.0101^b^** |
| Cervical Carcinoma | CC | 1 | 0 | 0 | 1.15% | 0.00% | 0.00% | >0.9999^a^ | 0.4579^a^ |
| Melanoma | MC | 1 | 0 | 0 | 0.63% | 0.00% | 0.00% | 0.2423^a^ | 0.1215^a^ |
| Soft Tissue Sarcoma | STS | 3 | 1 | 2 | 0.37% | 0.17% | 0.36% | 0.8561^c^ | 0.6859^c^ |
| Others | Others | 4 | 7 | 31 | 0.78% | 0.75% | 0.98% | 0.9352^b^ | 0.6662^b^ |
| Pancreatic Adenocarcinoma | PAAD | 1 | 1 | 4 | 0.32% | 0.19% | 0.20% | 0.7061^c^ | 0.8129^c^ |
| Urothelial Carcinoma | UC | 2 | 6 | 27 | 1.08% | 1.55% | 2.33% | 0.6571^b^ | 0.2772^b^ |
| Chondrosarcoma | CHOS | 1 | 0 | 0 | 9.09% | 0.00% | N/A | 0.2292^a^ | N/A |

^a^ Fisher's exact test; ^b/c^ Chi-square test or Yates' continuity corrected chi-square test; ^*^ Significant differences between Chinese and MSKCC cohorts; N/A, not available
