# Supplementary material for: Illuminating the FGFR fusion landscape in Chinese patients: unveiling novel molecular insights and clinical implications
Source: Oncologist. 2025 Oct 14;30(11):oyaf347. doi: 10.1093/oncolo/oyaf347 (PMC12640125; doi:10.1093/oncolo/oyaf347)
Supplement: oyaf347_Supplementary_Data [file oyaf347_supplementary_data.zip › Supplementary table S2.docx]

**Supplementary Table S2. Proportion of *FGFR1/2/3* rearrangements across different cancer types in Chinese and MSKCC cohorts**

| **Cancer type** | **Abbreviation** | **Chinese cohort** | | | **MSKCC cohort (2017)** | | | **MSKCC cohort (2021)** | | |
| --- | --- | --- | --- | --- | --- | --- | --- | --- | --- | --- |
|  |  | ***FGFR1*** | ***FGFR2*** | ***FGFR3*** | ***FGFR1*** | ***FGFR2*** | ***FGFR3*** | ***FGFR1*** | ***FGFR2*** | ***FGFR3*** |
| Glioma | GBM | 7.14% | 4.29% | 91.43% | 0.00% | 0.00% | 100.00% | N/A | N/A | N/A |
| Head and Neck Carcinoma | HNC | 33.33% | 0.00% | 66.70% | 0.00% | 0.00% | 100.00% | 0.00% | 0.00% | 100.00% |
| Lung Carcinoma | LC | 30.77% | 15.38% | 53.85% | 0.00% | 25.00% | 75.00% | 17.65% | 23.53% | 58.82% |
| Breast Carcinoma | BRCA | 0.00% | 0.00% | 0.00% | 0.00% | 0.00% | 100.00% | 12.50% | 62.50% | 25.00% |
| Gastric Cancer | GC | 0.00% | 50.00% | 50.00% | 0.00% | 0.00% | 0.00% | 33.33% | 66.67% | 0.00% |
| Colorectal Carcinoma | CRC | 33.33% | 66.67% | 0.00% | 100.00% | 0.00% | 0.00% | 46.15% | 38.46% | 15.38% |
| Bile Duct Carcinoma | BDC | 16.67% | 75.00% | 8.33% | 0.00% | 100.00% | 0.00% | 0.00% | 98.44% | 1.56% |
| Hepatocellular Carcinoma | HCC | 0.00% | 100.00% | 0.00% | 0.00% | 0.00% | 0.00% | 0.00% | 0.00% | 0.00% |
| Kidney Renal Clear Cell Carcinoma | KIRC | 0.00% | 100.00% | 0.00% | 0.00% | 0.00% | 0.00% | 0.00% | 0.00% | 0.00% |
| Prostate Adenocarcinoma | PRAD | 0.00% | 0.00% | 0.00% | 100.00% | 0.00% | 0.00% | 42.86% | 14.29% | 42.86% |
| Endometrial Carcinoma | EC | 0.00% | 50.00% | 50.00% | 0.00% | 0.00% | 100.00% | 0.00% | 28.57% | 71.43% |
| Cervical Carcinoma | CC | 0.00% | 0.00% | 100.00% | 0.00% | 0.00% | 0.00% | 0.00% | 0.00% | 0.00% |
| Melanoma | MC | 0.00% | 0.00% | 100.00% | 0.00% | 0.00% | 0.00% | 0.00% | 0.00% | 0.00% |
| Soft Tissue Sarcoma | STS | 100.00% | 0.00% | 0.00% | 0.00% | 100.00% | 0.00% | 50.00% | 0.00% | 50.00% |
| Others | Others | 0.00% | 25.00% | 75.00% | 0.00% | 14.29% | 85.71% | 0.00% | 16.13% | 83.87% |
| Pancreatic Adenocarcinoma | PAAD | 0.00% | 100.00% | 0.00% | 0.00% | 100.00% | 0.00% | 0.00% | 100.00% | 0.00% |
| Urothelial Carcinoma | UC | 0.00% | 0.00% | 100.00% | 0.00% | 0.00% | 100.00% | 0.00% | 3.70% | 96.30% |
| Chondrosarcoma | CHOS | 0.00% | 0.00% | 100.00% | 0.00% | 0.00% | 0.00% | N/A | N/A | N/A |

N/A, not available
