# Supplementary material for: Illuminating the FGFR fusion landscape in Chinese patients: unveiling novel molecular insights and clinical implications
Source: Oncologist. 2025 Oct 14;30(11):oyaf347. doi: 10.1093/oncolo/oyaf347 (PMC12640125; doi:10.1093/oncolo/oyaf347)
Supplement: oyaf347_Supplementary_Data [file oyaf347_supplementary_data.zip › Supplementary table S3.docx]

**Supplementary Table S3. Uncommon partners of *FGFR1-3* rearrangements by DNA-NGS in this study**

| **Patients ID** | **Cancer type** | **Uncommon fusion** | **Position1** | **Position2** |
| --- | --- | --- | --- | --- |
| case 1^&^ | CRC | *BCR-FGFR1* | Int1 | Int1 |
| case 2^&^ | BDC | *CNTN3-FGFR1*^ | Int3 | Int17 |
| case 3 | LC | *FGFR1-HMG20A*^ | Int2 | Int3 |
| case 4 | STS | *FGFR1-HOOK3* | Int17 | Int4 |
| case 2^&^ | BDC | *FGFR1-MTUS1* | Int17 | Int9 |
| case 5 | GBM | *FGFR1-PCM1* | Ex18 | Int8 |
| case 6 | HNC | *FGFR1-PLAG1* | Int1 | Int1 |
| case 7 | STS | *FGFR1-PSMG2*^ | Int5 | Int2 |
| case 8 | STS | *FGFR1-RAB11FIP1*^#^ | Ex18 | Int5 |
| case 9 | LC | *intergenic-FGFR1* | * | Int7 |
| case 10 | LC | *intergenic-FGFR1* | * | Ex8 |
| case 11 | LC | *KCNU1-FGFR1* | Int2 | Int3 |
| case 16 | GBM | *FGFR2-AFAP1L2* | Int17 | Int1 |
| case 1^&^ | CRC | *FGFR2-CASP7* | Int17 | Int7 |
| case 17 | BDC | *FGFR2-CCDC125*^ | Int17 | Int5 |
| case 18 | KIRC | *FGFR2-GMNN*^ | Int17 | Int2 |
| case 19 | GC | *FGFR2-intergenic* | Int17 | * |
| case 20 | LC | *FGFR2-intergenic* | Int17 | * |
| case 21 | GBM | *FGFR2-KIF11*^ | Int17 | Int8 |
| case 22 | BDC | *FGFR2-NRBF2* | Int17 | Int1 |
| case 23 | GC | *FGFR2-PDE2A* | Int17 | Int6 |
| case 24 | PAAD | *FGFR2-PKD2L1*^ | Int17 | Int12 |
| case 25 | BDC | *FGFR2-SCLT1*^ | Int17 | Int2 |
| case 26 | EC | *FGFR2-VCL*^#^ | Int17 | Int1 |
| case 37^&^ | GBM | *FGFR3-ACOT7*^ | Ex10 | Int7 |
| case 38 | GBM | *FGFR3-AFF4*^ | Int17 | Int11 |
| case 39 | GBM | *FGFR3-BAIAP2* | Ex18 | Int1 |
| case 37^&^ | GBM | *FGFR3-CKAP5* | Int17 | Int24 |
| case 40 | MC | *FGFR3-intergenic* | Int17 | * |
| case 41 | BDC | *FGFR3-ITGA9*^ | Ex18 | Int27 |
| case 42 | GBM | *FGFR3-MCRIP1*^ | Ex18 | Int1 |
| case 43 | GC | *FGFR3-PHTF2* | Ex18 | Int10 |
| case 44 | GBM | *FGFR3-POC1A* | Int17 | Int7 |
| case 45 | GBM | *FGFR3-TMPO*^ | Ex18 | Int1 |
| case 46 | LC | *FGFR3-VEGFB*^ | 3'UTR | Ex5 |
| case 47 | LC | *MAEA-FGFR3*^ | Int5 | Int7 |

^&^ One patient has two *FGFR* fusions; ^*^ Breakpoints of *FGFR* fusion partners were located in intergenic region; ^^^ Novel fusion partners detected by DNA-NGS in our cohort; ^#^ Uncommon fusion partners with a total number of 2 shared by our cohort and MSKCC 2021/2017 cohorts; BDC: Bile Duct Carcinoma; CRC: Colorectal Carcinoma; EC: Endometrial Carcinoma; GBM: Glioma; GC: Gastric Cancer; HNC: Head and Neck KIRC: Kidney Renal Clear Cell Carcinoma; LC: Lung Carcinoma; MC: Melanoma; PAAD: Pancreatic Adenocarcinoma; STS: Soft Tissue Sarcoma
