# Supplementary material for: Illuminating the FGFR fusion landscape in Chinese patients: unveiling novel molecular insights and clinical implications
Source: Oncologist. 2025 Oct 14;30(11):oyaf347. doi: 10.1093/oncolo/oyaf347 (PMC12640125; doi:10.1093/oncolo/oyaf347)
Supplement: oyaf347_Supplementary_Data [file oyaf347_supplementary_data.zip › Supplementary table S4.docx]

**Supplementary Table S4. Chromosome distribution of *FGFR1/2/3* rearrangements by DNA-NGS in our cohort**

| **Fusion** | **Gene 1** | **Chr 1** | **Gene 2** | **Chr 2** | **Intra/Inter-chromosome** | **Partner type** | **Count** |
| --- | --- | --- | --- | --- | --- | --- | --- |
| *FGFR1-CNTN3* | *CNTN3* | chr3 | *FGFR1* | chr8 | Inter-chromosome | Novel | 1 |
| *FGFR1-HOOK3* | *FGFR1* | chr8 | *HOOK3* | chr8 | Intra-chromosome | Uncommon | 1 |
| *intergenic-FGFR1* | *intergenic* | chr8 | *FGFR1* | chr8 | Intra-chromosome | Uncommon | 2 |
| *KCNU1-FGFR1* | *KCNU1* | chr8 | *FGFR1* | chr8 | Intra-chromosome | Uncommon | 1 |
| *FGFR1-MTUS1* | *FGFR1* | chr8 | *MTUS1* | chr8 | Intra-chromosome | Uncommon | 1 |
| *FGFR1-PCM1* | *FGFR1* | chr8 | *PCM1* | chr8 | Intra-chromosome | Uncommon | 1 |
| *FGFR1-PLAG1* | *FGFR1* | chr8 | *PLAG1* | chr8 | Intra-chromosome | Uncommon | 1 |
| *FGFR1-RAB11FIP1* | *FGFR1* | chr8 | *RAB11FIP1* | chr8 | Intra-chromosome | Uncommon | 1 |
| *FGFR1-TACC1* | *FGFR1* | chr8 | *TACC1* | chr8 | Intra-chromosome | Common | 4 |
| *FGFR1-HMG20A* | *FGFR1* | chr8 | *HMG20A* | chr15 | Inter-chromosome | Novel | 1 |
| *FGFR1-PSMG2* | *FGFR1* | chr8 | *PSMG2* | chr18 | Inter-chromosome | Novel | 1 |
| *BCR-FGFR1* | *BCR* | chr22 | *FGFR1* | chr8 | Inter-chromosome | Uncommon | 1 |
| *FGFR2-SCLT1* | *FGFR2* | chr10 | *SCLT1* | chr4 | Inter-chromosome | Novel | 1 |
| *FGFR2-CCDC125* | *FGFR2* | chr10 | *CCDC125* | chr5 | Inter-chromosome | Novel | 1 |
| *FGFR2-GMNN* | *FGFR2* | chr10 | *GMNN* | chr6 | Inter-chromosome | Novel | 1 |
| *FGFR2-AFAP1L2* | *FGFR2* | chr10 | *AFAP1L2* | chr10 | Intra-chromosome | Uncommon | 1 |
| *FGFR2-BICC1* | *FGFR2* | chr10 | *BICC1* | chr10 | Intra-chromosome | Common | 5 |
| *FGFR2-CASP7* | *FGFR2* | chr10 | *CASP7* | chr10 | Intra-chromosome | Uncommon | 1 |
| *FGFR2-CCDC6* | *FGFR2* | chr10 | *CCDC6* | chr10 | Intra-chromosome | Common | 2 |
| *FGFR2-intergenic* | *FGFR2* | chr10 | *intergenic* | chr10 | Intra-chromosome | Uncommon | 2 |
| *FGFR2-KIAA1217* | *FGFR2* | chr10 | *KIAA1217* | chr10 | Intra-chromosome | Common | 1 |
| *FGFR2-KIF11* | *FGFR2* | chr10 | *KIF11* | chr10 | Intra-chromosome | Novel | 1 |
| *FGFR2-NRBF2* | *FGFR2* | chr10 | *NRBF2* | chr10 | Intra-chromosome | Uncommon | 1 |
| *FGFR2-PKD2L1* | *FGFR2* | chr10 | *PKD2L1* | chr10 | Intra-chromosome | Novel | 1 |
| *FGFR2-SHTN1* | *FGFR2* | chr10 | *SHTN1* | chr10 | Intra-chromosome | Common | 1 |
| *FGFR2-TACC2* | *FGFR2* | chr10 | *TACC2* | chr10 | Intra-chromosome | Common | 1 |
| *FGFR2-VCL* | *FGFR2* | chr10 | *VCL* | chr10 | Intra-chromosome | Uncommon | 1 |
| *FGFR2-PDE2A* | *FGFR2* | chr10 | *PDE2A* | chr11 | Inter-chromosome | Uncommon | 1 |
| *FGFR3-ACOT7* | *FGFR3* | chr4 | *ACOT7* | chr1 | Inter-chromosome | Novel | 1 |
| *FGFR3-ITGA9* | *FGFR3* | chr4 | *ITGA9* | chr3 | Inter-chromosome | Novel | 1 |
| *FGFR3-POC1A* | *FGFR3* | chr4 | *POC1A* | chr3 | Inter-chromosome | Uncommon | 1 |
| *FGFR3-intergenic* | *FGFR3* | chr4 | *intergenic* | chr4 | Intra-chromosome | Uncommon | 1 |
| *MAEA-FGFR3* | *MAEA* | chr4 | *FGFR3* | chr4 | Intra-chromosome | Novel | 1 |
| *FGFR3-NSD2* | *FGFR3* | chr4 | *NSD2* | chr4 | Intra-chromosome | Common | 1 |
| *FGFR3-TACC3* | *FGFR3* | chr4 | *TACC3* | chr4 | Intra-chromosome | Common | 67 |
| *FGFR3-AFF4* | *FGFR3* | chr4 | *AFF4* | chr5 | Inter-chromosome | Novel | 1 |
| *FGFR3-PHTF2* | *FGFR3* | chr4 | *PHTF2* | chr7 | Inter-chromosome | Uncommon | 1 |
| *FGFR3-CKAP5* | *FGFR3* | chr4 | *CKAP5* | chr11 | Inter-chromosome | Uncommon | 1 |
| *FGFR3-VEGFB* | *FGFR3* | chr4 | *VEGFB* | chr11 | Inter-chromosome | Novel | 1 |
| *FGFR3-TMPO* | *FGFR3* | chr4 | *TMPO* | chr12 | Inter-chromosome | Novel | 1 |
| *FGFR3-BAIAP2* | *FGFR3* | chr4 | *BAIAP2* | chr17 | Inter-chromosome | Uncommon | 1 |
| *FGFR3-MCRIP1* | *FGFR3* | chr4 | *MCRIP1* | chr17 | Inter-chromosome | Novel | 1 |
