# Supplementary material for: Illuminating the FGFR fusion landscape in Chinese patients: unveiling novel molecular insights and clinical implications
Source: Oncologist. 2025 Oct 14;30(11):oyaf347. doi: 10.1093/oncolo/oyaf347 (PMC12640125; doi:10.1093/oncolo/oyaf347)
Supplement: oyaf347_Supplementary_Data [file oyaf347_supplementary_data.zip › Supplementary table S5.docx]

**Supplementary Table S5. Coordinates and Coverage Summary of Intronic Bait Probes Targeting *FGFR1*, *FGFR2*, *FGFR3* and *FGFR4* in the DNA Panel**

| **Genes** | **NM** | **Intron** | **Kinase Domains** | **chr** | **start** | **end** | **strand** |
| --- | --- | --- | --- | --- | --- | --- | --- |
| *FGFR1* | NM_001174063 | intron1 | Ex11-18 | 8 | 38315053 | 38325497 | - |
| *FGFR1* | NM_001174063 | intron2 | Ex11-18 | 8 | 38287467 | 38314872 | - |
| *FGFR1* | NM_001174063 | intron9 | Ex11-18 | 8 | 38275892 | 38277055 | - |
| *FGFR1* | NM_001174063 | intron10 | Ex11-18 | 8 | 38275510 | 38275744 | - |
| *FGFR1* | NM_001174063 | intron17 | Ex11-18 | 8 | 38271323 | 38271434 | - |
| *FGFR2* | NM_000141 | intron1 | Ex11_18 | 10 | 123353482 | 123357474 | - |
| *FGFR2* | NM_000141 | intron10 | Ex11_18 | 10 | 123260462 | 123263302 | - |
| *FGFR2* | NM_000141 | intron16 | Ex11_18 | 10 | 123243318 | 123244907 | - |
| *FGFR2* | NM_000141 | intron17 | Ex11_18 | 10 | 123239536 | 123243210 | - |
| *FGFR3* | NM_000142 | intron10 | Ex11_18 | 4 | 1806697 | 1807080 | + |
| *FGFR3* | NM_000142 | intron17 | Ex11_18 | 4 | 1808662 | 1808841 | + |
| *FGFR4* | NM_022963 | intron8 | Ex9_16 | 5 | 176520553 | 176520653 | + |
