# Supplementary material for: Illuminating the FGFR fusion landscape in Chinese patients: unveiling novel molecular insights and clinical implications
Source: Oncologist. 2025 Oct 14;30(11):oyaf347. doi: 10.1093/oncolo/oyaf347 (PMC12640125; doi:10.1093/oncolo/oyaf347)
Supplement: oyaf347_Supplementary_Data [file oyaf347_supplementary_data.zip › Supplementary table S6.docx]

**Supplementary Table S6. Chromosomal location for *FGFR1/2/3* rare fusions identified by DNA/RNA-based NGS**

| **Patient (ID)** | **Rare fusion type** | **DNA-NGS data** | | **RNA-NGS data** | |
| --- | --- | --- | --- | --- | --- |
|  |  | **Fusions** | **Chromosome position** | **Fusions** | **Chromosome position** |
| case 9 | Intergenic-breakpoint fusions | *intergenic-FGFR1* | Chr8:75827973-Chr8:38279577 | Negative | Negative |
| case 10 |  | *intergenic-FGFR1* | Chr8:36245361-Chr8:38279358 | Negative | Negative |
| case 19 |  | *FGFR2-intergenic* | Chr10:123242196-Chr10:123394107 | N/A | N/A |
| case 20 |  | *FGFR2-intergenic* | Chr10:123243196-Chr10:120222060 | *FGFR2-PLEKHA4*^b^ | Chr10:123243212-Chr19:49357521 |
| case 40 |  | *FGFR3-intergenic* | Chr4:1808832-Chr4:2758335 | *FGFR3-TNIP2* | Chr4:1808661-Chr4:2749672 |
| case 3 | Novel fusions | *FGFR1-HMG20A* | Chr8:38299451-Chr15:77751008 | Negative | Negative |
| case 7 |  | *FGFR1-PSMG2* | Chr8:38284110-Chr18:12707020 | Negative | Negative |
| case 37 |  | *FGFR3-ACOT7* | Chr4:1806618-Chr1:6344947 | Negative | Negative |
| case 46 |  | *FGFR3-VEGFB* | Chr4:1810080-Chr11:64004669 | Negative | Negative |
| case 25 |  | *FGFR2-SCLT1* | Chr10:123241671-Chr4:129999626 | *FGFR2-SCLT1* | Chr10:123243212-Chr4:129965206 |
| case 21 |  | *FGFR2-KIF11* | Chr10:123242531-Chr10:94375696 | *FGFR2-KIF11* | Chr10:123243212-Chr10:94376494 |
| case 38 |  | *FGFR3-AFF4* | Chr4:1808670-Chr5:132230737 | *FGFR3-AFF4* | Chr4:1808661-Chr5:132228810 |
| case 41 |  | *FGFR3-ITGA9* | Chr4:1808917-Chr3:37855172 | *FGFR3-ITGA9* | Chr4:1808661-Chr3:37860382 |
| case 45 |  | *FGFR3-TMPO* | Chr4:1808979-Chr12:98911183 | *FGFR3-TMPO* | Chr4:1808661-Chr12:98921664 |
| case 2 |  | *CNTN3-FGFR1* | Chr3:74543853-Chr8:38271360 | N/A | N/A |
| case 17 |  | *FGFR2-CCDC125* | Chr10:123242472-Chr5:68602850 | N/A | N/A |
| case 18 |  | *FGFR2-GMNN* | Chr10:123240837-Chr6:24780658 | N/A | N/A |
| case 24 |  | *FGFR2-PKD2L1* | Chr10:123242850-Chr10:102050503 | N/A | N/A |
| case 47 |  | *MAEA-FGFR3* | Chr4:1325817-Chr4:1804651 | N/A | N/A |
| case 42 |  | *FGFR3-MCRIP1* | Chr4:1808863-Chr17:79789667 | N/A | N/A |

^b^ Novel fusion was identified by RNA-NGS

N/A: not available.
