# Supplementary figures and images for: Illuminating the FGFR fusion landscape in Chinese patients: unveiling novel molecular insights and clinical implications
Source: Oncologist. 2025 Oct 14;30(11):oyaf347. doi: 10.1093/oncolo/oyaf347 (PMC12640125; doi:10.1093/oncolo/oyaf347)

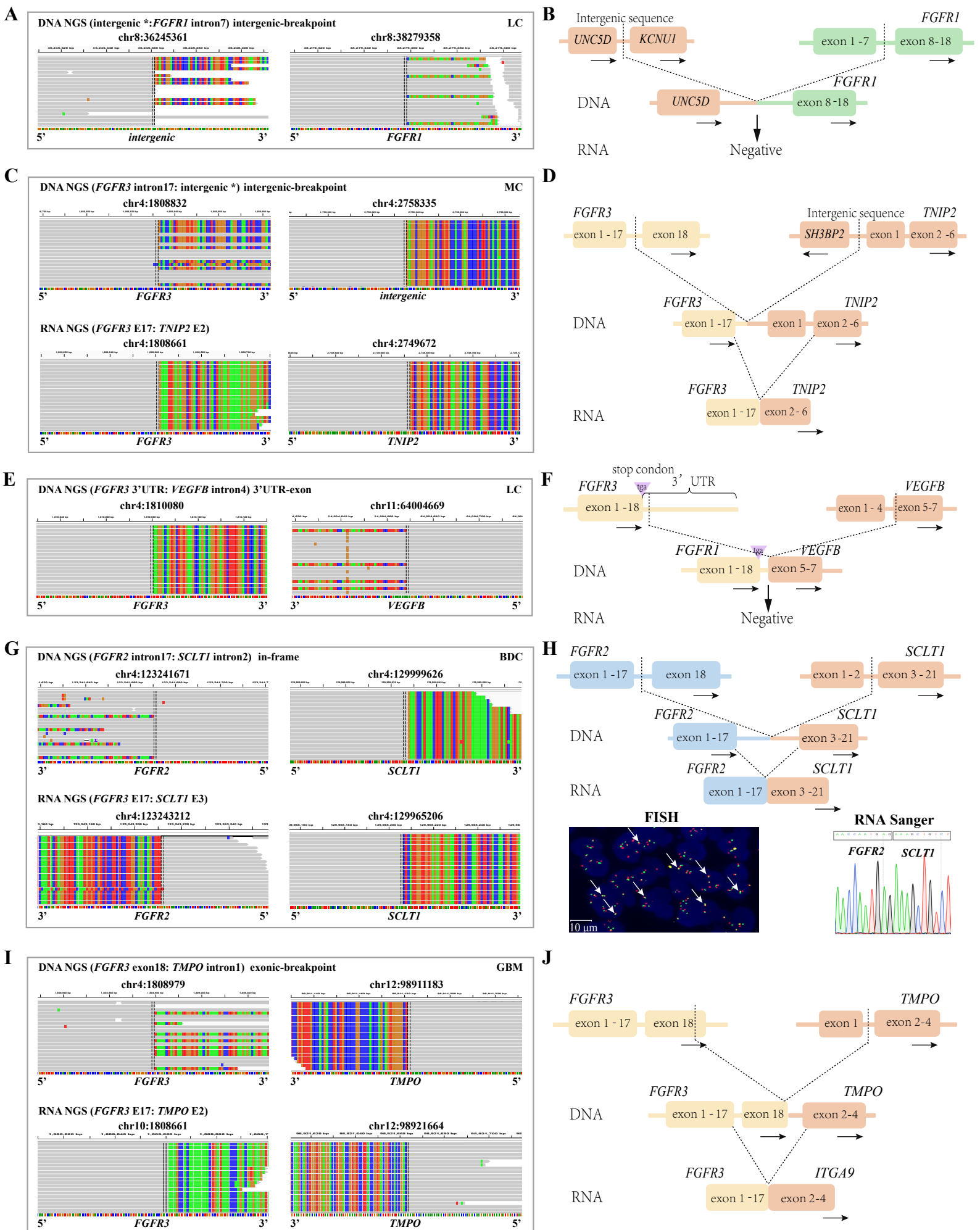

Supplement: oyaf347_Supplementary_Data [file oyaf347_supplementary_data.zip › Fig. S4.pdf]

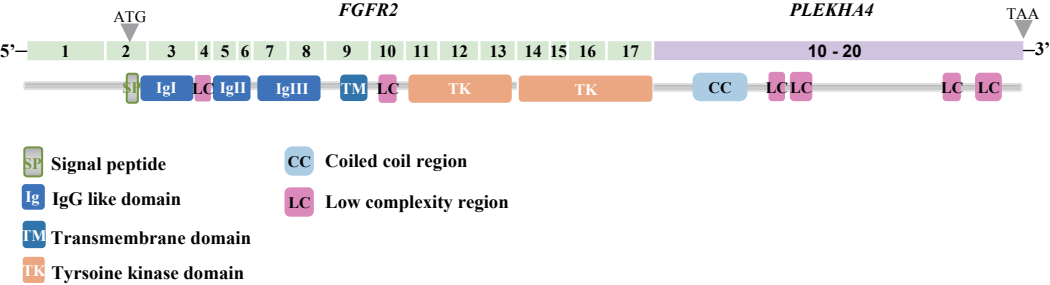

Supplement: oyaf347_Supplementary_Data [file oyaf347_supplementary_data.zip › Fig. S6.pdf]

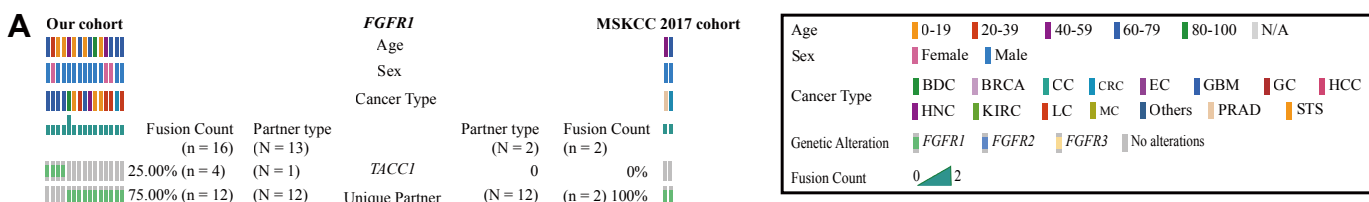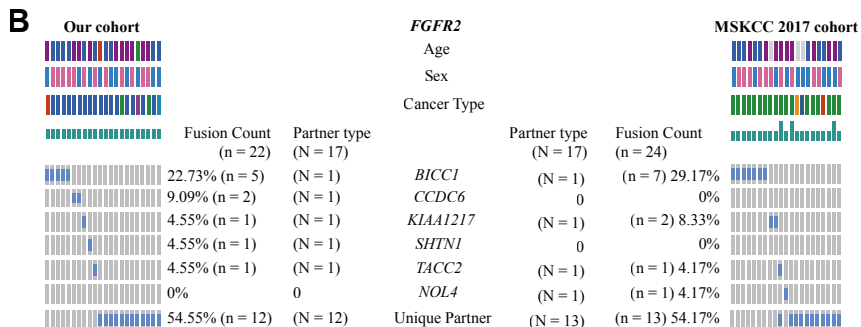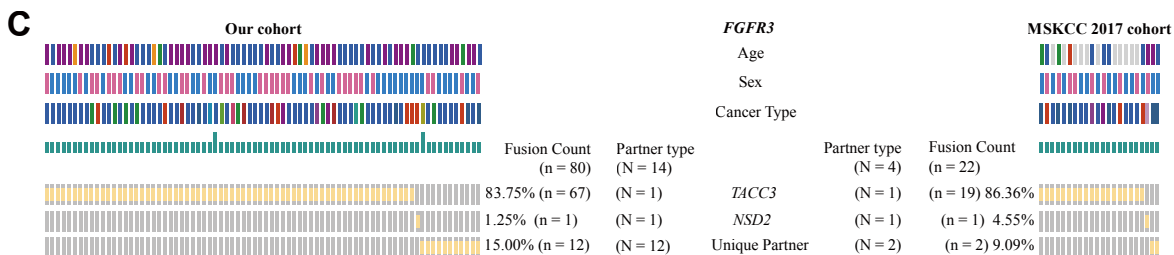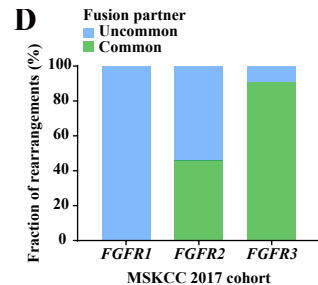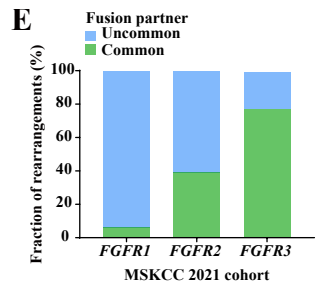

Supplement: oyaf347_Supplementary_Data [file oyaf347_supplementary_data.zip › Supplementary Fig. S1.pdf]

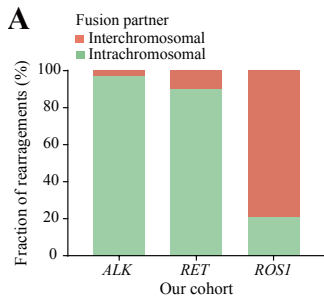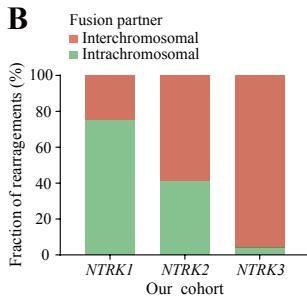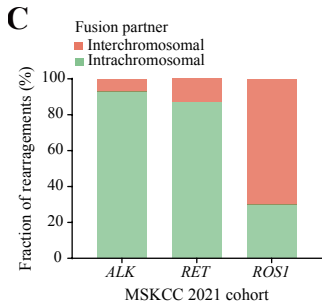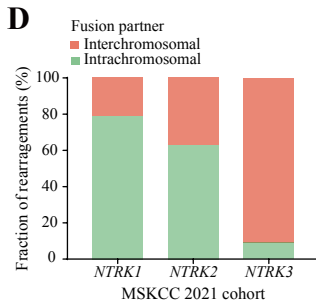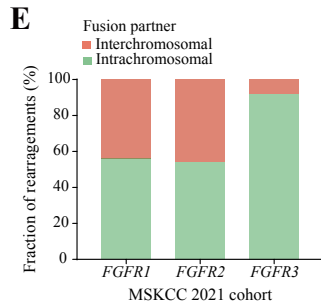

Supplement: oyaf347_Supplementary_Data [file oyaf347_supplementary_data.zip › Supplementary Fig. S2.pdf]

**A**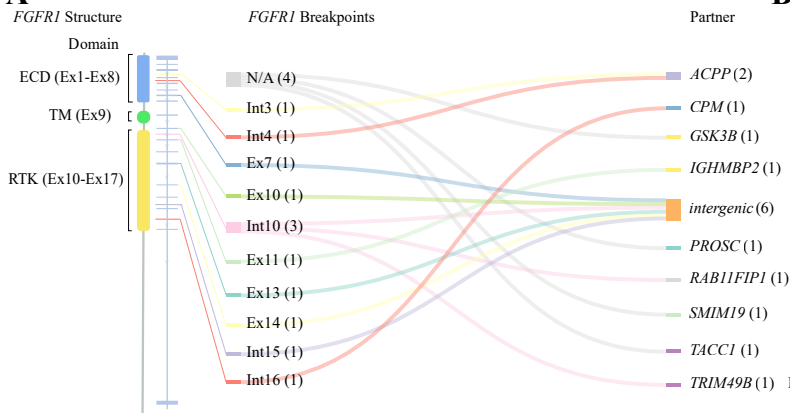**B**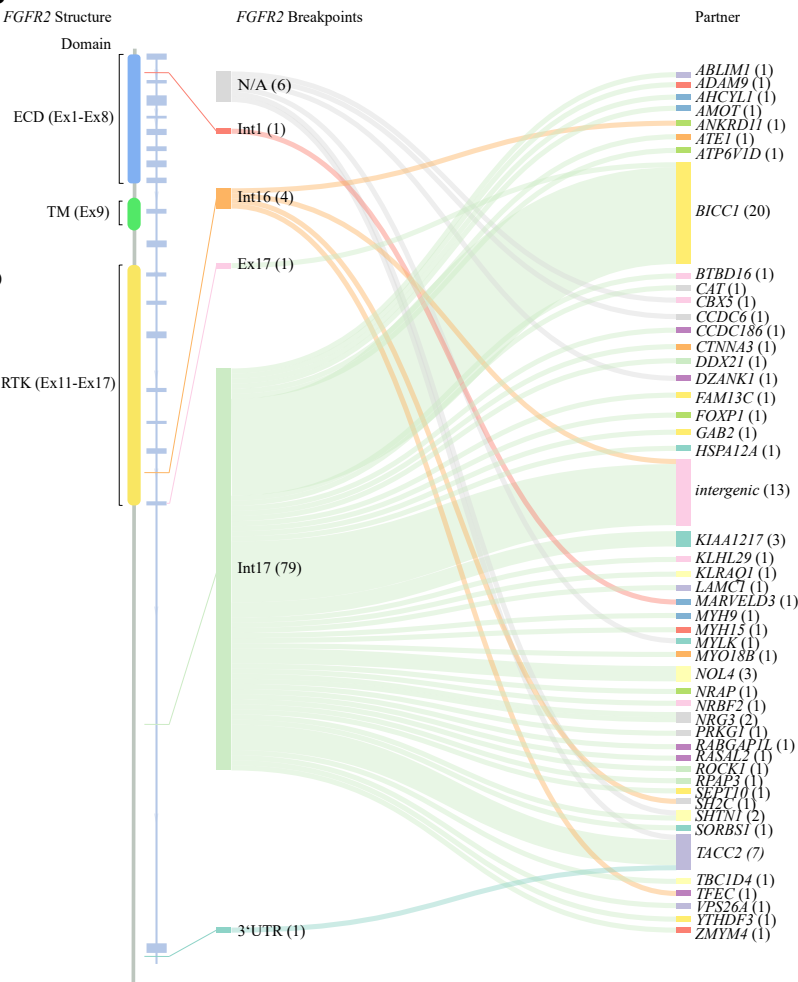**C**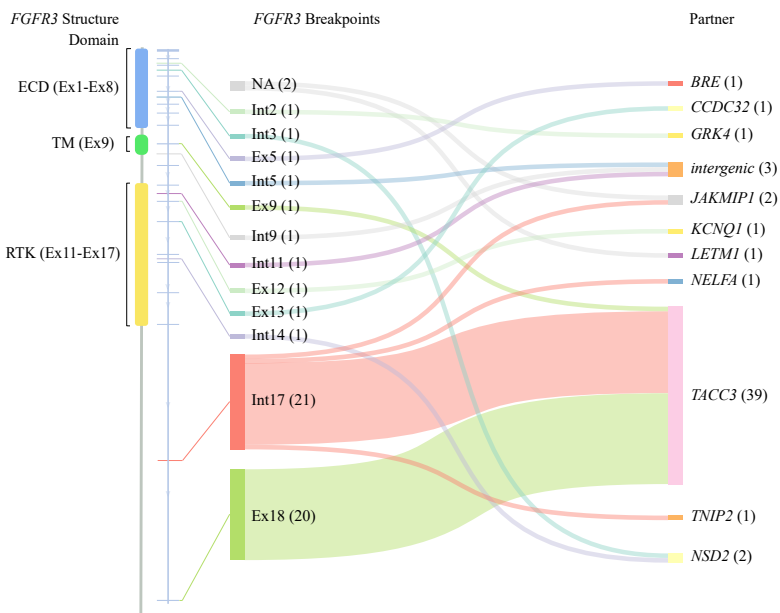

Supplement: oyaf347_Supplementary_Data [file oyaf347_supplementary_data.zip › Supplementary Fig. S3.pdf]

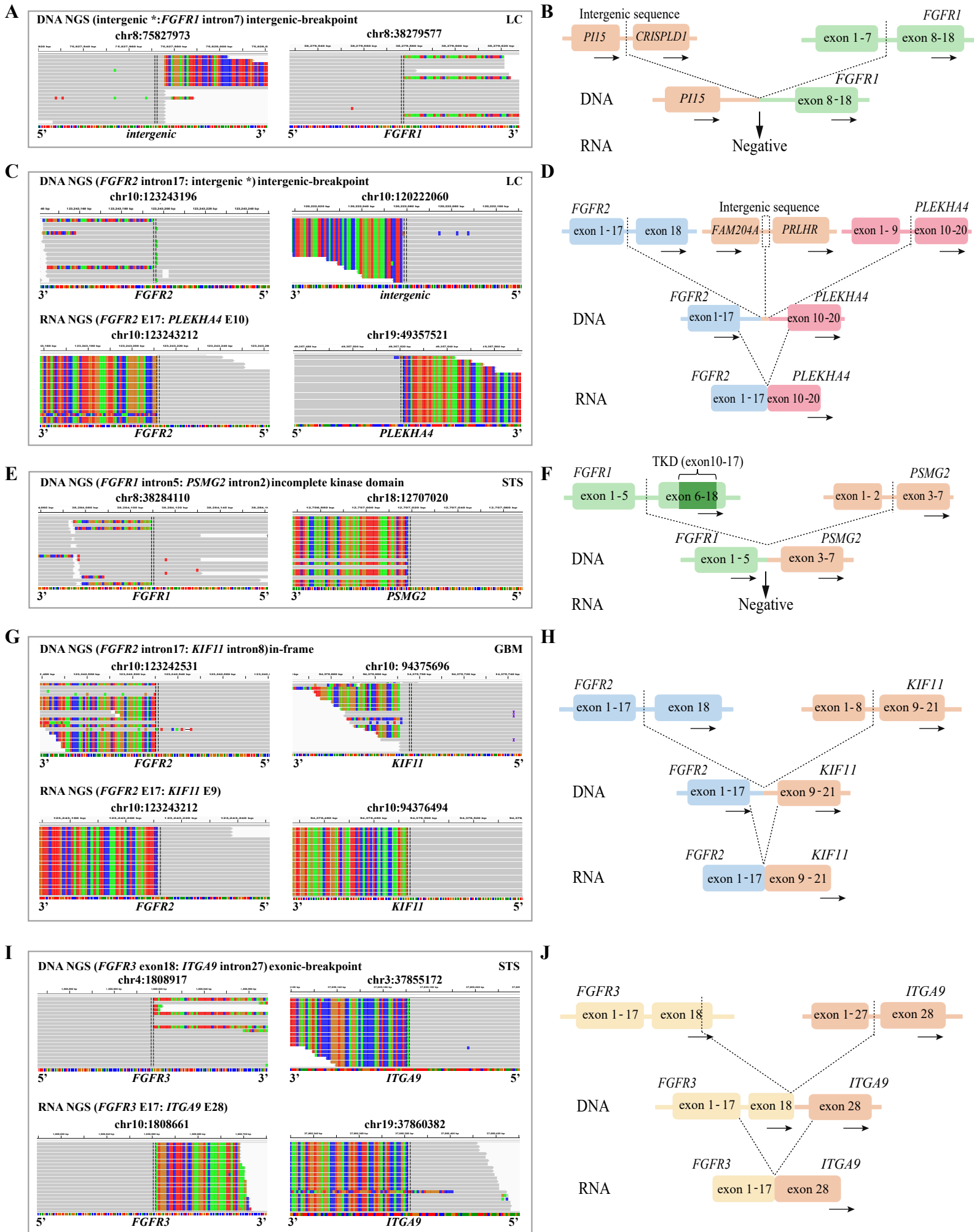

Supplement: oyaf347_Supplementary_Data [file oyaf347_supplementary_data.zip › Supplementary Fig. S5.pdf]
